# Immune-mediated adverse events following atezolizumab and bevacizumab in a multinational Latin American cohort of unresectable hepatocellular carcinoma

**DOI:** 10.18632/oncotarget.28721

**Published:** 2025-05-19

**Authors:** Leonardo Gomes da Fonseca, Federico Piñero, Margarita Anders, Carla Bermudez, Ezequiel Demirdjian, Adriana Varón, Daniela Perez, Jorge Rodriguez, Oscar Beltrán, Ezequiel Ridruejo, Pablo Caballini, Alexandre Araujo, Juan Diego Torres Florez, Juan Ignacio Marín, Marina Villa, Federico Orozco, Jaime Poniachik, Sebastián Marciano, Fernando Bessone, Manuel Mendizabal

**Affiliations:** ^1^Instituto do Cancer do Estado de São Paulo, Hospital das Clínicas, Universidade São Paulo, Brazil; ^2^Hepatology and Liver Transplant Unit, Hospital Universitario Austral, Argentina; ^3^Department of Hepatology, Hospital Alemán, Argentina; ^4^Department of Hepatology and Liver Transplantation, Hospital Italiano de Buenos Aires, Argentina; ^5^Department of Liver Transplantation, Sanatorio Sagrado Corazón, Argentina; ^6^Department of Hepatology, Fundación Cardioinfantil, Colombia; ^7^Department of Gastroenterology, Hospital Padilla, Tucumán, Argentina; ^8^Department of Liver Transplantation, Hospital Central de Mendoza, Argentina; ^9^Department of Gastroenterology, Hospital Clínico de la Universidad de Chile, Chile; ^10^Department of Hepatology, Centro de Educación Médica e Investigaciones Clínicas (CEMIC), Argentina; ^11^Department of Gastroenterology, Hospital Centenario de Rosario, Santa Fe, Argentina; ^12^Department of Gastroenterology, Hospital das Clínicas de Porto Alegre, Universidade Federal do Rio Grande do Sul, Brazil; ^13^Department of Gastroenterology, Hospital Universitario Fundación Santa Fe de Bogotá, Colombia; ^14^Department of Hepatology and Liver Transplantation, Hospital Pablo Tobón Uribe, Medellín, Colombia; ^15^Department of Internal Medicine, Hospital Comarcal de Blanes, Córdoba, Argentina; ^*^Co-first authorship

**Keywords:** liver cancer, immunotherapy, adverse events, immunology, real-world

## Abstract

Aims: Latin America has been underrepresented in trials evaluating immunotherapy for hepatocellular carcinoma (HCC). We aimed to describe the incidence of immune-related adverse events (irAEs) and their impact on outcomes in a Latin American cohort.

Methods: A multicenter prospective study was conducted in Argentina, Brazil, Chile, and Colombia, including patients who received atezolizumab plus bevacizumab. A time-covarite proportional hazard analysis evaluated the effect of irAEs.

Results: 99 patients were included. The median treatment duration was 6 months, with a median survival of 17.0 months (95% CI: 12.6–19.8). The irAE incidence rate was 2.1 cases per 100 persons-months (cumulative incidence 18.1% (95% CI: 11.1–27.2%)). Median time to irAE was 2.3 months (range 1.4–4.8), most frequently hepatitis (*n* = 6), thyroiditis (*n* = 5), and 8/18 required steroids. Follow-up, treatment duration, and overall survival were similar regardless of the occurrence of irAEs (HR = 1.71, 95% CI: 0.76–3.86; *P* = 0.19). Baseline alpha-feto protein ≥400 ng/ml (HR: 2.9 (95% CI: 1.1–7.6)) was independently associated with irAE.

Conclusion: The incidence of irAEs in this cohort is lower than reported in controlled trials, withouut impact on survival outcomes. Education and early recognition are crucial to ensure that these events are identified and addressed.

## INTRODUCTION

Globally, hepatocellular carcinoma (HCC) remains a leading cause of cancer-related deaths. The high lethality of HCC is driven by the concomitance of cirrhosis and its complications in most patients, alongside the high proportion of patients diagnosed at an advanced stage when no curative-intend treatment is feasible [1, 2]. Advancements have been made in the management of advanced-stage disease with novel systemic therapies, leading to improved tumor control and survival [3].

Over the last few years, clinical trials proved the superiority of immunotherapy-based combinations over multikinase inhibitors. Atezolizumab, a monoclonal Ig4 antibody against programmed death receptor (anti-PD-L1), plus bevacizumab, a monoclonal antibody targeting the vascular endothelial growth factor (anti-VEGF), were the first treatment to show superiority over sorafenib in a phase III trial and received regulatory approval across different countries [4].

The strict inclusion criteria hamper translating the results of clinical trials to daily practice. In the case of HCC, clinical trials predominantly included patients with well-preserved liver function, favorable performance status, no significant comorbidities, and a lower risk of competitive mortality risks [5]. Moreover, patients from Latin America are underrepresented in clinical trials, and regional validation is needed.

It is well established that although immunotherapy improves outcomes in HCC patients, they are complicated by immune-related adverse events (irAEs). The incidence of irAEs may vary by regional context, patient selection, treatment parameters, and local practices. There is growing recognition that irAEs can affect treatment compliance, quality of life, and survival [6–9]. In a dual cohort study, patients developing immune-mediated hepatitis have paradoxically shown increased irAE incidence rates, with a higher proportion of spontaneous resolution, without the need for high-dose steroids [10]. However, reproducible conclusions have been limited by the fact that real-world patients are more likely to have comorbidities, reduced liver function reserve, and, thus, risk of liver decompensation upon the occurrence of adverse events. Therefore, the present study investigated the incidence, type, severity, and management of irAEs and their potential association with survival in a region where these data have scantily been reported.

## RESULTS

Of 306 BCLC A-C patients receiving systemic therapy over the study period, 32.3% (*n* = 99) received atezolizumab plus bevacizumab (*n* = 91 as first-line and *n* = 8 as second-line treatment). Table 1 describes the characteristics of the included population. Of these, 70.7% were enrolled from Argentina, 15.2% from Colombia, and 14.1% from Brazil. The primary associated etiology was metabolic-associated steatotic liver disease (MASLD) in 31.3%, followed by chronic hepatitis C in 30.3%, and alcohol-related liver disease in 9.1%. Most patients had cirrhosis (82.3%), and 19.5% had presented prior decompensation, including 10.1% with mild ascites at treatment initiation. Regarding baseline staging, 70.7% were classified as BCLC-C and 29.3% as BCLC-B. In addition, 38.4% had macrovascular invasion (Vp1-Vp4), and 33.3% had extrahepatic spread. Most patients had Child-Pugh A (81.8%) and ECOG-performance status 0–1 (95.7%).

**Table 1:** Characteristics of patients treated with Atezo + Bev (*n* = 99)

Variables	*n* = 99 (15.8%)
**Age**, years (±SD)	67 ± 9
**Male gender**, *n* (%)	78 (78.8)
**Obesity**, *n* (%)	18 (18.9)
**Comorbidities**, *n* (%)	58 (58.6)
**Etiology of liver disease**, *n* (%)	
Viral/non-viral	38 (38.4)/61 (61.6)
Hepatitis C	30 (30.3)
Hepatitis B	8 (8.1)
Metabolic associated steatotic liver disease	31 (31.3)
Alcoholic liver disease	9 (9.1)
Other etiologies	21 (21.2)
**Cirrhosis**, *n* (%)	82 (82.3)
**Child Pugh A/B**, *n* (%)	64 (78.1)/18 (21.9)
**Prior decompensation**, *n* (%)	16/82 (19.5)
**Median total Bilirubin**, mg/dl (IQR)	0.9 (0.6-1.4)
**Median Albumin**, g/dl (IQR)	3.8 (3.3-4.1)
**Median INR,** (IQR)	1.0 (1.0-1.2)
**Median ALBI score,** (IQR)	−2.42 (−2.83; −1.91)
**ALBI grade 1**, *n* (%)	32 (32.3)
**ALBI grade 2**, *n* (%)	45 (45.4)
**ALBI grade 3**, *n* (%)	22 (22.2)
**Mild Ascites**, *n* (%)	10 (10.1)
**Hepatic Encephalopathy grades I–II**, *n* (%)	2 (2.0)
**ECOG 0/1**, *n* (%)	95 (95.7)/4 (4.3)
**Median serum AFP**, ng/ml (IQR)	32.0 (5.0–844)
**AFP ≥100 ng/ml**, *n* (%)	31 (31.3)
**AFP ≥400 ng/ml**, *n* (%)	23 (23.2)
**Macrovascular tumor invasion**, *n* (%)	38 (38.4)
**Metastatic disease**, *n* (%)	33 (33.3)
**BCLC**, *n* (%)	
**B**	29 (29.3)
**C**	70 (70.7)

Abbreviations: HCC: hepatocellular carcinoma; AFP: alpha-fetoprotein; IQR: interquartile range.

### Treatment characteristics and outcomes

**Figure 1:**
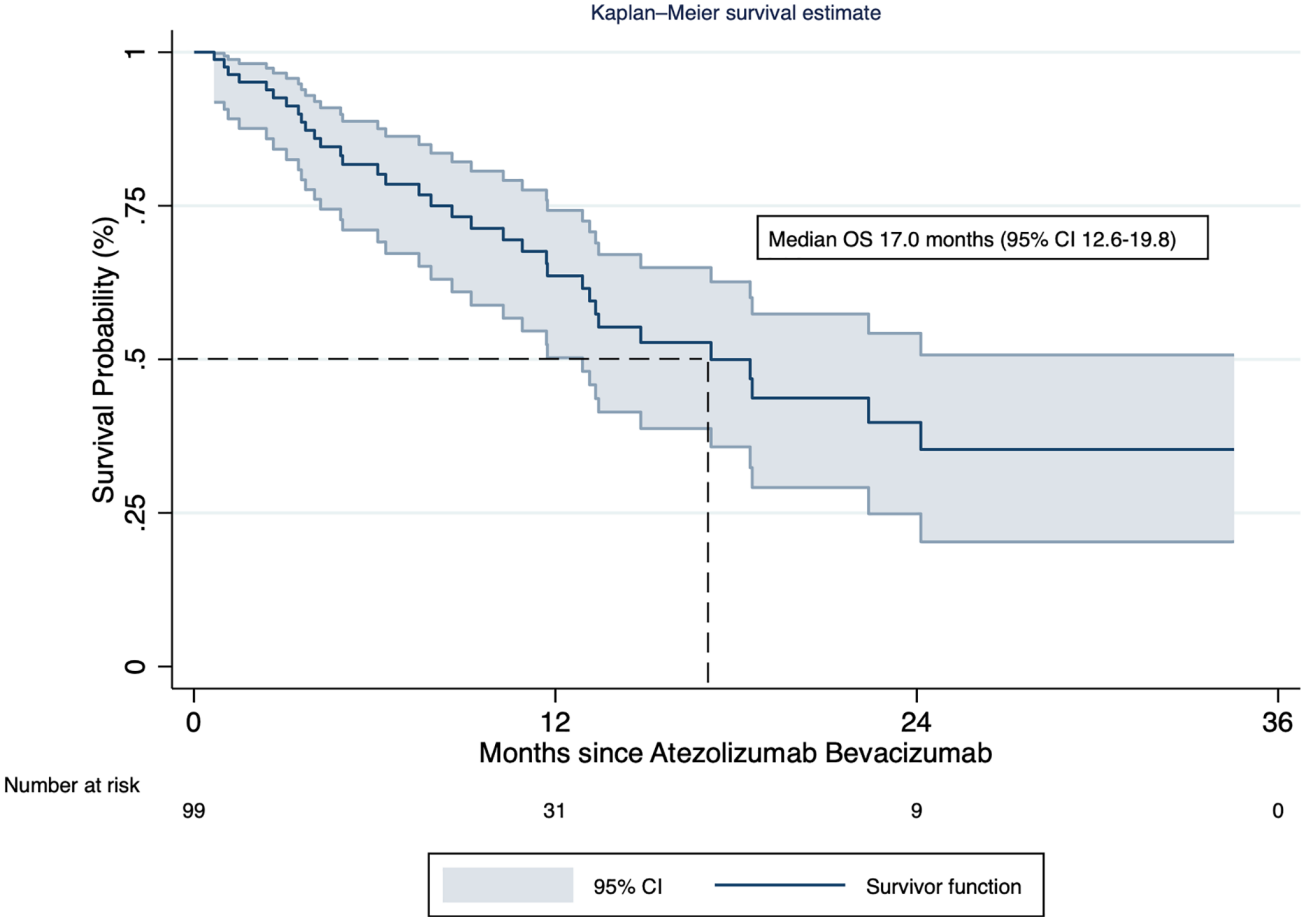
Kaplan-Meier survival curve of the patients treated with atezolizumab plus bevacizumab.

Atezolizumab plus bevacizumab median treatment duration was six months (interquartile range (IQR): 2.6–14.6) with a median of 5 cycles (IQR: 3–11.5). After a median follow-up of 7.7 months (IQR: 4.5–17.2), the median overall survival since treatment initiation was 17.0 months (95% CI: 12.6–19.8). The 12, 18, and 24 months survival rates were 61.4% (95% CI: 47.8–72.4%), 43.1% (95% CI: 28.5–56.9%), and 34.8% (95% CI: 19.9–50.2%), respectively (Figure 1).

After multivariate analysis, patients with prior decompensation showed an increased risk of death (HR: 3.83; 95% CI: 1.34–10.95; *p* < 0.012), adjusted for Child-Pugh B vs. A (HR = 2.62; 95% CI: 1.05–6.57; *p* = 0.039), and baseline AFP ≥400 ng/ml (HR = 4.01, 95% CI: 1.73–9.29; *P* = 0.001). Noteworthy, the median overall survival of patients with Child-Pugh B was 8.6 months (95% CI: 2.63-not reached) versus 18.5 months (95% CI: 13.33-not reached) for Child-Pugh A patients (Table 2).

**Table 2:** Prognostic variables in patients with Atezo Bev. Cox regression analysis

Variable	irAEs *n* = 18 (18.1%)	Without irAEs *n* = 81 (81.2%)	*P*-values
**Age**, years (± SD)	68 ± 8	67 ± 9	0.62
**Gender, Male**, *n* (%)	15 (83.3)	63 (77.8)	0.44
**Obesity**, *n* (%)	4 (23.5)	14 (17.9)	0.41
**Comorbidities**, *n* (%)	10 (55.6)	48 (59.3)	0.48
**Cirrhosis**, *n* (%)	16 (88.9)	66 (81.5)	0.35
**Etiology of liver disease**, *n* (%)			
Viral/non-viral	9 (50.0)/9 (50.0)	29 (35.8)/52 (64.2)	0.20
Hepatitis C	8 (44.4)	22 (27.2)	0.12
Metabolic associated steatotic liver disease	4 (22.2)	27 (33.3)	0.27
Alcoholic liver disease	–	9 (11.1)	0.10
**Child Pugh A/B**, *n* (%)	14 (77.8)/4 (22.2)	67 (82.7)/14 (17.3)	0.42
**Prior decompensation**, *n* (%)	–	16 (24.2)	0.02
**ECOG 0–1**, *n* (%)	18 (100)	77 (95.1)	0.44
**Median total Bilirubin**, mg/dl (IQR)	0.8 (0.7–1.6)	0.9 (0.6–1.3)	0.66
**Median Albumin**, g/dl (IQR)	3.8 (3.6–4.2)	3.8 (3.3–4.1)	0.51
**Median INR,** (IQR)	1.1 (1.0–1.2)	1.0 (1.0–-1.2)	0.82
**Median ALBI score, (IQR)**	−2.45 (−2.95; −2.11)	−2.39 (−2.82; −1.91)	0.69
**ALBI grade 1, *n* (%)**	7 (38.9)	25 (30.9)	0.50
**ALBI grade 2, *n* (%)**	9 (50.0)	36 (44.4)	
**ALBI grade 3, *n* (%)**	2 (11.1)	20 (24.7)	
**Median serum AFP**, ng/ml (IQR)	150.7 (5.4–1624.9)	28.5 (4.9–487.7)	0.30
**AFP ≥100 ng/ml**, *n* (%)	9 (50.0)	22 (27.2)	0.06
**AFP ≥400 ng/ml**, *n* (%)	7 (38.9)	16 (19.7)	0.08
**Macrovascular tumor invasion**, *n* (%)	6 (33.3)	32 (39.5)	0.42
**Metastatic disease**, *n* (%)	8 (44.4)	25 (30.9)	0.20
**BCLC**, *n* (%)			
**B**	5 (27.8)	24 (29.6)	0.56
**C**	13 (72.2)	57 (70.4)	

Normal Values: Alpha-fetoprotein 0.6–4.4 ng/ml. Abbreviations: AFP: Alpha-fetoprotein; HCC: Hepatocellular carcinoma; HR: hazard ratio; MASLD: Metabolic associated steatotic liver disease.

Temporary treatment interruptions were necessary in 23 (23.2%) patients. A total of 58 patients (58.6%) experienced adverse events of any grade, 27 (46.5%) classified as mild, 7 (12.1%) as moderate, and 24 (41.4%) as severe. The most frequent adverse events were fatigue (22%), hypertension (12.1%) and bleeding (11.1%). Supplementary Table 1 describes the adverse events reported.

The most common reasons for treatment discontinuation were progressive disease (24.2%), worsening liver function (11.1%), treatment intolerance (9.1%), and symptomatic progression (3.0%). The median survival after atezolizumab plus bevacizumab discontinuation was 2.6 months (95% CI: 1.2–3.4).

### Immune-related adverse events and their impact on survival

**Figure 2:**
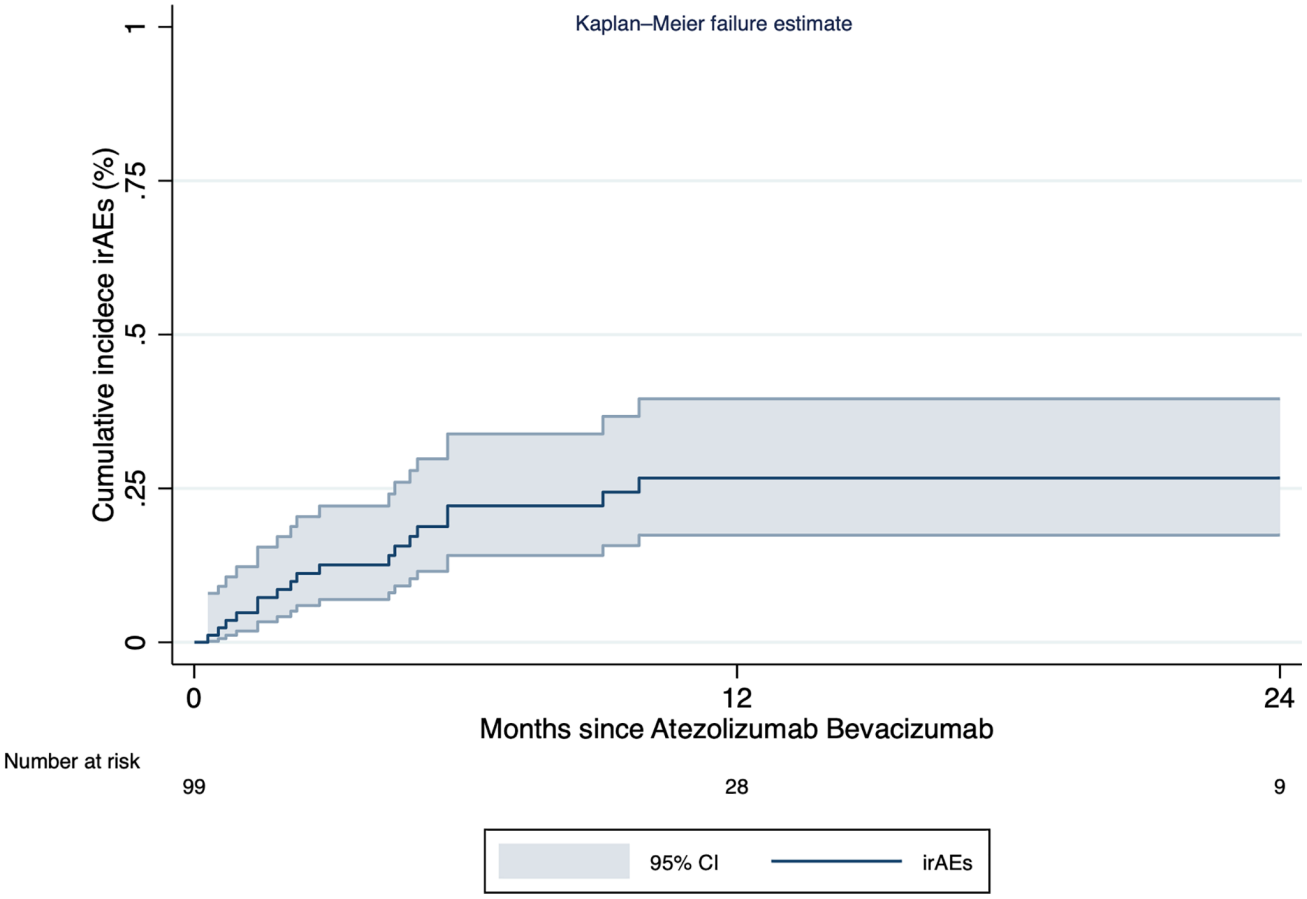
Cumulative incidence curve of immune related adverse events in patients treated with atezolizumab plus bevacizumab.

Eighteen patients presented irAEs according to the investigator´s judgment with an incidence rate of 2.1 cases per 100 persons-months (cumulative incidence 18.1% (95% CI: 11.1–27.2%)). The median time to irAE was 2.3 months (IQR 1.4–4.8) (Figure 2). The most frequently reported irAEs were hepatitis (*n* = 6), thyroiditis (*n* = 5), and nephritis (*n* = 3). Twelve events required medical treatment, eight required steroids, and four other immunosuppressants. A complete resolution of the irAEs was described in 9 (50%) events with a median time to resolution of 30 days (IQR: 14–48). Four (22.2%) patients were rechallenged with immunotherapy, and two patients presented recurrence of irAE after rechallenging, with a median time to recurrence of 3.5 months (IQR: 0–7).

The median follow-up was similar regardless of the occurrence of irAEs. For patients presenting irAEs, the median follow-up was 5.4 months (IQR: 3.7–11.7) and 8.5 months (IQR: 4.1–16.9) (*P* = 0.27) for those without irAEs. Accordingly, treatment duration was similar between both groups (5.1 months, IQR = 1.4–10.0; versus 6.5 months, IQR = 2.7–16.9, *P* = 0.14). No significant differences regarding demographics and baseline features were observed between patients developing and not developing irAEs. (Table 3).

**Table 3:** Comparison between patients with and without immune-related adverse events associated with Atezolizumab (*n* = 99)

Variable	Unadjusted HR (95% CI)	*P*	Adjusted HR (95% CI)	*P*
**Age**, years	0.99 (0.96−1.03)	0.77		
**Male gender**	0.66 (0.30−1.47)	0.31		
**Cirrhosis**	2.95 (0.89−9.71)	0.07		
**Prior decompensation**	3.01 (1.17−7.71)	0.02	3.83 (1.34–10.95)	0.012
**Etiology of liver disease**				
Viral/non-viral	0.92 (0.47−1.81)	0.81		
Hepatitis C	1.07 (0.53−2.17)	0.85		
MASLD	1.01 (0.46−2.25)	0.97		
**Number of HCC nodules**	1.03 (0.91−1.18)	0.61		
**Diffuse intrahepatic pattern**	2.06 (0.85−5.0)	0.11		
**Major nodule diameter (mm)**	1.01 (0.99−1.02)	0.12		
**Serum bilirubin**, mg/dl	1.53 (1.15−2.0)	0.003		
**Albumin**, gr/dl	0.52 (0.29−0.94)	0.03		
**Child Pugh B vs. A**	3.08 (1.33−7.12)	0.009	2.62 (1.05−6.57)	0.039
**ALBI grade 1 vs. grades 2-3**	1.79 (0.84−3.86)	(0.13)		
**Serum AFP**	1.01 (1.01−1.02)	0.043		
**AFP ≥400 ng/ml**	3.66 (1.75−7.65)	0.001	4.01 (1.73−9.29)	0.001
**Macrovascular tumor invasion**	1.54 (0.78−3.03)	0.21		
**Metastatic disease**	1.11 (0.56−2.23)	0.76		
**BCLC C vs. B**	1.64 (0.74−3.63)	0.22		

Abbreviations: HCC: hepatocellular carcinoma; AFP: alpha-fetoprotein; IQR: interquartile range; INR: international normalized ratio.

**Figure 3:**
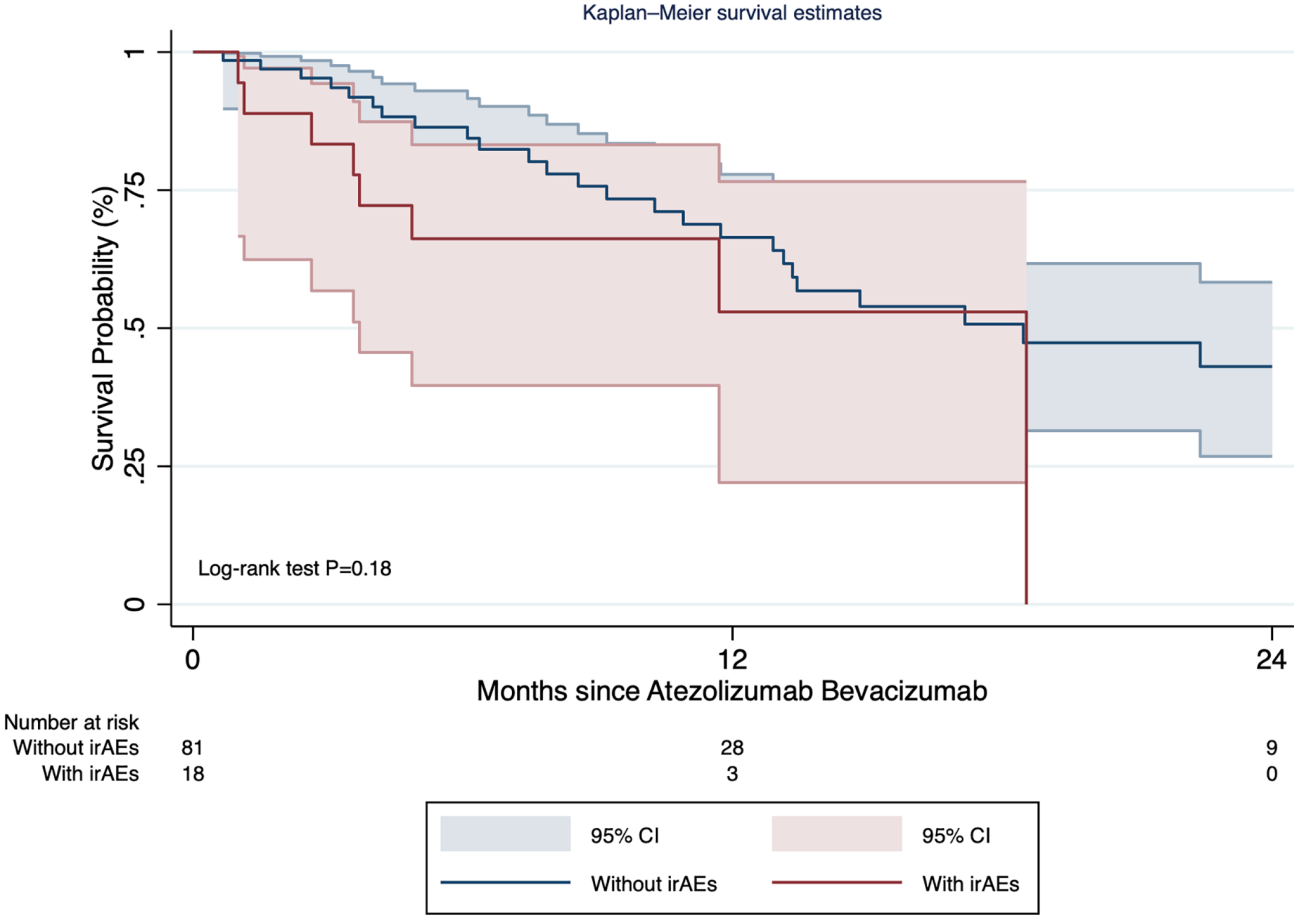
Survival curves of patients with and without immune related adverse events.

The median overall survival of patients who presented irAEs was 18.5 months (95% CI: 3.7–not reached) since treatment initiation, while the median overall survival of patients who did not present irAEs was 18.5 months (95% CI: 12.9–not reached). The occurrence of irAEs was not statistically associated with survival since treatment initiation (HR = 1.71, 95% CI: 0.76–3.86; *P* = 0.19) (Figure 3), and its effect was not time-covariate dependent (HR = 0.66; 95% CI: 0.25–1.72; *p* = 0.16). Considering the subgroup with irAEs, the median overall survival post-irAE was 2.9 months (95% CI: 0.2–9.57).

There were 27.3% of patients (*n* = 27) with grade I adverse events, 7.1% with grade II (*n* = 7), and 24.4% grades III or higher (*n* = 24). No impact on survival of irAEs grade II or higher was observed (HR: 1.30; 95% CI: 0.66–2.57; 0 = 0.45).

### Predictive factors for irAEs occurrence

We examined potential predictive factors for irAE development by evaluating baseline characteristics. In the univariate analysis, AFP≥ 400 ng/ml and diffuse intrahepatic were significantly associated with irAEs occurrence (at a cutoff of *p* < 0.10). In the multivariate analysis, AFP ≥400 ng/ml remained significantly associated with the irAEs, with an HR = 2.92 (95% CI: 2.22–7.60); *P* = 0.03. (Table 4).

**Table 4:** Predictive factors for irAEs development. Cox regression analysis

Variable	Unadjusted HR (95% CI)	*P*	Adjusted HR (95% CI)	*P*
**Age**, years	1.01 (0.96−1.06)	0.68		
**Male gender**	1.21 (0.35−4.22)	0.76		
**Cirrhosis**	2.86 (0.87−9.42)	0.084		
**Etiology of liver disease**				
Viral/non-viral	1.35 (0.53−3.4)	0.52		
Hepatitis C	1.61 (0.63−4.07)	0.32		
MASLD	0.84 (0.28−2.56)	0.76		
**Number of HCC nodules**	0.95 (0.77−1.18)	0.66		
**Diffuse intrahepatic pattern**	2.55 (0.84−7.75)	0.09	2.54 (0.83−7.7)	0.10
**Major nodule diameter (mm)**	1.00 (0.99−1.02)	0.41		
**Serum bilirubin**, mg/dl	1.23 (0.81−1.88)	0.32		
**Albumin**, gr/dl	1.31 (0.52−3.34)	0.57		
**Child Pugh B vs. A**	1.61 (0.53−4.92)	0.40		
**Serum AFP**	1.00 (0.99−1.00)	0.61		
**AFP ≥400 ng/ml**	2.93 (1.12−7.7)	0.03	2.92 (1.11−7.6)	0.03
**Macrovascular tumor invasion**	0.88 (0.33−2.35)	0.80		
**Metastatic disease**	1.52 (0.60−3.86)	0.38		
**BCLC C vs. B**	1.16 (0.41−3.28)	0.77		

Normal Values: Alpha-fetoprotein 0.6–4.4 ng/ml. Abbreviations: AFP: Alpha-fetoprotein; HCC: Hepatocellular carcinoma; HR: hazard ratio; BCLC: Barcelona Clinic Liver Cancer; ECOG: Eastern Cooperative Oncology Group; MASLD: Metabolic associated steatotic liver disease.

## DISCUSSION

This study provides valuable insights into the incidence and impact of irAEs in patients with HCC treated with the combination of atezolizumab and bevacizumab in real-world practice. As immune checkpoint inhibitors continue to gain relevance in the treatment of advanced HCC, understanding the nuances of irAE development and their consequences on patient outcomes is critical, particularly in populations underrepresented in clinical trials with a higher prevalence of cirrhosis and other comorbidities.

The incidence rate of irAEs in our cohort, at 2.1 cases per 100 person-months and a cumulative incidence of 18.1%, aligns with previous reports of irAEs in immunotherapy-treated patients [4, 11, 12], although it may still be underreported. This is likely because mild irAEs that do not significantly impact the clinical course or lead to treatment discontinuation may go unnoticed or underreported in real-world settings. For example, we observed a few dermatologic events such as pruritus or rash, which can have a less prominent impact on quality of life.

Interestingly, while irAEs are often viewed as a marker of immune activation and potentially improved outcomes in cancer therapy, our analysis did not demonstrate a significant survival benefit in patients who developed irAEs compared to those who did not. Other studies found divergent results. For instance, Ng et al. showed median OS for grade ≥3 irAE versus grades 1–2 irAE versus no irAE of 26.9 versus 14.0 versus 4.6 months [12]. Similarly, Xu et al. observed that the median OS and disease control rate was better in patients with irAEs [11]. This lack of consistency across different cohorts may be attributed to several factors, including the relatively small sample size, diverse methodology in reporting irAEs, and heterogeneous median follow-up time. Further studies with larger cohorts and longer follow-up may be required to clarify this relationship.

The IMbrave150 trial reported that 68.7% of patients developed atezolizumab-related adverse events [4]. However, this study registered adverse events other than irAEs. In particular, the frequency of hepatitis was markedly different from that in our study; 43.2% of patients in the IMbrave150 study developed hepatitis, whereas around 6% of our patient’s developed hepatitis. In a multicentric prospective cohort, Celsa et al. observed an incidence of 11.4% of immune-related liver injury in patients with HCC, with a resolution of 72.1%. The incidence was lower in patients with other solid tumors, at 2.6% [10]. Hepatitis is a relevant adverse event due to the risk of liver decompensation. Besides, hepatitis can be related to the HCC or caused by concomitant drugs or alcohol use. The lower incidence of irAEs, including hepatitis, observed in our cohort compared to the IMbrave150 trial and other published studies may be attributable to several factors. First, differences in patient selection may play a role; clinical trials often include patients with more favorable baseline characteristics and stricter inclusion criteria, while our real-world cohort likely reflects a more heterogeneous population. Second, monitoring intensity and frequency in routine clinical practice may be less rigorous than in controlled trial settings. Third, mild increments on liver function enzymes have been reported on clinical trials, but not all these elevations were defined as clinical hepatitis. The difference between clinical trials and real-world data can be attributed to the trial´s requirement for registering minimal alterations and a stricter monitoring schedule in research protocols. Finally, variations in adverse event definitions and documentation standards across centers may contribute to underestimation, particularly for laboratory-based toxicities such as asymptomatic hepatitis.

Notably, baseline alpha-fetoprotein (AFP) values ≥400 ng/ml were significantly associated with the development of irAEs. Elevated AFP levels have been previously linked to more aggressive HCC biology and worse prognosis [13], but their role in predicting irAEs is novel. Interestingly, a recent cohort observed that patients with liver injury using immunotherapy had higher levels of AFP compared to patients without liver injury [10]. These findings suggest that patients with high AFP may require closer monitoring for potential immune-related toxicities.

While AFP is commonly used as a prognostic biomarker in HCC and is often associated with more aggressive tumor biology and advanced disease stage, its role in modulating immune response is not recognized. One possible explanation is that higher AFP levels reflect an underlying pro-inflammatory tumor microenvironment, increased tumor antigenicity or tumor necrosis, which could potentiate immune activation and response to immunotherapy. This may also increase susceptibility to immune-related toxicity. Alternatively, patients with elevated AFP may have received more intensive monitoring or laboratory testing, increasing the likelihood of irAE detection. It is also important to consider the potential for confounding. Patients with advanced disease and higher AFP levels might be more prone to progression and hepatic dysfunction, which could mimic the appearance of irAEs. Although multivariable modeling was used to adjust for known confounders, residual confounding cannot be excluded in a real-world cohort of this size. All these hypotheses warrant further exploration and validation in future studies.

The relatively short post-irAE survival of 2.9 months indicates the potential severity of irAEs in this patient population, where the majority present with underlying cirrhosis. Hepatitis was the most common irAE, with a substantial proportion of patients requiring corticosteroid treatment, underscoring the importance of timely recognition and management of these toxicities. Given the hepatic reserve limitations in patients with cirrhosis, irAEs such as hepatitis can significantly affect clinical outcomes, and therefore, a tailored, multidisciplinary approach is essential.

While the results of this study did not demonstrate a significant impact of irAEs on overall survival, they emphasize the complexity of managing immune-related toxicities in real-world HCC populations, especially in regions like Latin America, where access to specialized care and management may vary across different centers. Early identification and personalized management of irAEs remain critical in optimizing therapeutic outcomes, particularly in patients with advanced liver disease.

The strengths of this study include its multicenter prospective cohort design and the inclusion of a real-world patient population. However, limitations common in observational studies, such as the lack of a centralized definition of irAE assessment, potentially result in misclassification bias. However, it reflects the challenges encountered in clinical practice. Relatively short follow-up periods must be acknowledged, with a potential selection bias. The short median follow-up of 7.7 months might limit the ability to observe long-term outcomes, such as kidney and hematologic irAEs. This may lead to an underestimation of both irAE incidence and potential survival differences between patients with and without irAEs. Additionally, as immune-related toxicities can occur beyond the early treatment phase, longer observation would be necessary to assess their full spectrum and impact. Nevertheless, it should be noted that in the participating countries, approval of atezolizumab plus bevacizumab occurred at least 1–2 years after European, Asian and North American approval dates.

The short follow-up increases the impact of censored data due to ongoing follow-up, which may further affect the robustness of survival estimates. Future analyses with extended follow-up are warranted to validate these findings and better define the long-term safety and efficacy profile in this population.

The relatively small number of patients experiencing irAEs (*n* = 18) limits the statistical power of subgroup analyses and may affect the robustness of conclusions regarding the association between irAEs and survival outcomes. While we observed no statistically significant difference in overall survival between patients with and without irAEs (HR: 1.71, 95% CI: 0.76–3.86; *P* = 0.19), this analysis may be underpowered to detect a positive effect. The absence of a statistically significant association should be interpreted with caution and not considered definitive evidence of no effect. Larger cohorts or pooled analyses may be necessary to clarify the prognostic impact of irAEs in this population. Moreover, conducting further subgroup analysis, including the effect on survival stratifying each type of irAE would have led to further reduced sample power (e.g. hypothyroidism versus liver irAE, which in cirrhosis may have a different impact on survival). As the project continues to enroll and follow patients, it is expected that the power to detect new events will increase, potentially increasing the incidence of irAEs in this population.

In conclusion, the incidence of irAEs in this Latin American cohort aligned with real-world global data, highlighting the need for careful monitoring. The association between high AFP and irAEs requires further research to understand better the implications of irAEs on long-term outcomes in HCC patients. Ultimately, a multidisciplinary approach is crucial for managing irAEs to ensure that patients can continue to benefit from life-prolonging immunotherapies while minimizing adverse events.

## MATERIALS AND METHODS

### Study design, participating centers, and eligibility criteria

This observational multicenter prospective cohort study was conducted in 14 Latin American centers from Argentina, Chile, Brazil, and Colombia, including HCC patients from May 15th, 2018, to March 15th, 2024. The results shown in this report correspond to a pre-specified final analysis on April 1st, 2024, from a prospective cohort including Barcelona Clinic Liver Cancer (BCLC) stages A-C HCC patients treated with atezolizumab plus bevacizumab, either as first or second line systemic treatment [14].

In the present analysis, we included consecutive adult patients with clinical or histological diagnosis of HCC if all the following eligibility criteria were met: (1) Radiological, either with Computerized Axial Tomography (CT) or Magnetic Resonance Image (MRI), or histological diagnosis of HCC, according to international recommendations [15, 16]. (2) Locally advanced (non-surgical) or metastatic HCC, either BCLC A (treatment stage migration), B, or C at study enrollment. Patients could have been in other BCLC stages over their past medical history. (3) Received at least one dose of atezolizumab plus bevacizumab. Patients were excluded if other malignant tumors were present, presented with Child-Pugh C, BCLC-D, recent variceal bleeding, encephalopathy, prior systemic immunotherapy, enrollment in interventional clinical trials, incomplete baseline clinical data, concurrent active autoimmune diseases requiring immunosuppression or follow-up shorter than 30 days unless due to death.

The study protocol complied with international ethical statements and standards of Good Clinical Practice, requiring a signed informed consent and confidentiality agreement in all centers (CIE 18-078). Study data was registered on a web-based electronic case report from the Latin American Liver Research Education and Awareness Network (LALREAN, https://www.temasis.com.ar/lalrean-org). Results were reported according to STROBE guidelines [17].

### Data collection

Study variables included age, gender, demographic data, comorbid conditions, and etiology of liver disease. The tumor characteristics through imaging and serum alpha-feto protein values (AFP) were registered at HCC diagnosis and classified according to the BCLC staging systemic [3]. Performance status was described according to the Eastern Cooperative Oncology Group (ECOG) classification, and liver function was classified according to the Child-Pugh and ALBI (albumin-bilirubin) scores [18, 19]. Baseline laboratory values included platelet count, total bilirubin, international normalized ratio (INR), and serum albumin. We registered dates of atezolizumab plus bevacizumab initiation, definite suspension, and total number of treatment cycles.

### Immune-related adverse events

Local investigators were asked to register adverse events and irAEs that showed a causal relation with atezolizumab and bevacizumab. IrAEs were identified and diagnosed based on clinical, laboratory, and radiologic findings suggestive of immune-mediated toxicity, after exclusion of other etiologies such as infection or disease progression. Events were graded according to the *Common Terminology Criteria for Adverse Events (CTCAE) version 4.0*. To minimize inter-center variability, investigators were instructed to apply pre-specified definitions for each type of irAE, in alignment with CTCAE. To ensure consistency across participating sites, local investigators were contacted in case of inconsistencies, and suspected irAEs were reviewed and re-discussed when needed. IrAEs were categorized as thyroiditis, nephritis, pneumonitis, neurological, myositis, adrenal insufficiency, hepatitis, dermatitis, colitis, new-onset diabetes Mellitus (insulitis), carditis, pancreatitis and hypophysitis. Less commonly occurring irAEs were collectively categorized as “other.” Categorisation and management were generally based on the following recommendations: CTCAE grade 1, mild events not requiring systemic therapy or treatment delay; CTCAE grade 2, moderate events requiring symptomatic management or temporary interruption of immunotherapy, and CTCAE grade ≥3, severe events requiring hospitalization, or need for systemic corticosteroids or permanent discontinuation of systemic treatment [20]. The electronic database was reviewed centrally.

### Study endpoints

The primary endpoint of the study was to describe the incidence rates and cumulative incidence of irAE in patients treated with atezolizumab and bevacizumab. The secondary endpoints were to compare baseline characteristics between patients with or without irAEs, identify variables independently related to occurrence of irAEs and identify prognostic variables in this cohort of patients treated with atezolizumab plus bevacizumab.

### Statistical analysis

The incidence rates were calculated as the number of patients presenting at least one irAE per 100 persons-month, whereas the cumulative incidence is shown as cumulative rates and a 95% confidence interval (CI). The inverse Kaplan-Meier product and cumulative incidence curves for irAEs were estimated, being irAE the failure event (time to irAEs), and patients without irAEs were censored at the date of last follow-up or death.

Kaplan Meier survival curves were compared using the log-rank test (Mantel-Cox) from the date of atezolizumab plus bevacizumab initiation to the failure event or date of censoring. Cox proportional hazard analysis was conducted to evaluate independent variables associated with irAE. Follow-up and treatment duration times were compared between groups with or without irAEs. Multivariable Cox proportional hazards regression analyses were conducted to identify independently related variables with the outcome of interest, estimating hazard ratios (HR) and 95% CI. Variables from the crude analysis showing *P*-values <.10 were further included in the multivariable model in a step-by-step process, evaluating confounding effects (change in HR estimates more than 20%).

Survival analysis was conducted with death as the primary or failure event (date of death) and censored observations in the absence of death registered at the last date of follow-up. Overall survival was defined from the date of atezolizumab plus bevacizumab initiation until death or last follow-up (censored). We also estimated the HR of death in a multivariable Cox regression model. To address the effect of irAEs on survival, we evaluated its effect over time through a time-covariate analysis. Missing data were handled using a complete-case approach for primary analyses. For time-to-event analyses, censoring was applied at the last known follow-up date. Variables with >10% missingness were not included in multivariable models. Data quality and completeness were regularly audited across sites to ensure consistency. Data was analyzed using STATA 17.0 BE perpetual license (Stata, Texas, USA).

## SUPPLEMENTARY MATERIALS



## Abbreviations

HCC: hepatocellular carcinoma
irAE: immune-related adverse event
PD-L1: programmed death ligand
VEGF: vascular endothelial growth factor
BCLC: Barcelona Clinic Liver Cancer
CT: Computerized Tomography
MRI: magnetic resonance imaging
AFP: Alpha-fetoprotein
ECOG: Eastern Cooperative Oncology Group
ALBI: albumin-bilirubin
INR: international normalized ratio values
CTCA: common terminology criteria for adverse events
CI: Confidence Interval
HR: Hazard ratio
MASLD: metabolic associated steatotic liver disease
IQR: interquartile range
SD: standard deviation

## References

[1] Rumgay H , Arnold M , Ferlay J , Lesi O , Cabasag CJ , Vignat J , Laversanne M , McGlynn KA , Soerjomataram I . Global burden of primary liver cancer in 2020 and predictions to 2040. J Hepatol. 2022; 77:1598–606. 10.1016/j.jhep.2022.08.021. 36208844 PMC9670241

[2] Brar G , Greten TF , Graubard BI , McNeel TS , Petrick JL , McGlynn KA , Altekruse SF . Hepatocellular Carcinoma Survival by Etiology: A SEER-Medicare Database Analysis. Hepatol Commun. 2020; 4:1541–51. 10.1002/hep4.1564. 33024922 PMC7527688

[3] Reig M , Forner A , Rimola J , Ferrer-Fàbrega J , Burrel M , Garcia-Criado Á , Kelley RK , Galle PR , Mazzaferro V , Salem R , Sangro B , Singal AG , Vogel A , et al. BCLC strategy for prognosis prediction and treatment recommendation: The 2022 update. J Hepatol. 2022; 76:681–93. 10.1016/j.jhep.2021.11.018. 34801630 PMC8866082

[4] Finn RS , Qin S , Ikeda M , Galle PR , Ducreux M , Kim TY , Kudo M , Breder V , Merle P , Kaseb AO , Li D , Verret W , Xu DZ , et al, and IMbrave150 Investigators. Atezolizumab plus Bevacizumab in Unresectable Hepatocellular Carcinoma. N Engl J Med. 2020; 382:1894–905. 10.1056/NEJMoa1915745. 32402160

[5] Bruix J , da Fonseca LG , Reig M . Insights into the success and failure of systemic therapy for hepatocellular carcinoma. Nat Rev Gastroenterol Hepatol. 2019; 16:617–30. 10.1038/s41575-019-0179-x. 31371809

[6] Balaji A , Zhang J , Wills B , Marrone KA , Elmariah H , Yarchoan M , Zimmerman JW , Hajjir K , Venkatraman D , Armstrong DK , Laheru DA , Mehra R , Ho WJ , et al. Immune-Related Adverse Events Requiring Hospitalization: Spectrum of Toxicity, Treatment, and Outcomes. J Oncol Pract. 2019; 15:e825–34. 10.1200/JOP.18.00703. 31386608 PMC6743220

[7] Cluxton C , Naidoo J . Prospective Clinical Trials to Advance the Study of Immune Checkpoint Inhibitor Toxicity. Curr Oncol. 2023; 30:6862–71. 10.3390/curroncol30070502. 37504362 PMC10378048

[8] von Itzstein MS , Gerber DE , Bermas BL , Meara A . Acknowledging and addressing real-world challenges to treating immune-related adverse events. J Immunother Cancer. 2024; 12:e009540. 10.1136/jitc-2024-009540. 39038920 PMC11268023

[9] Lau G , Cheng AL , Sangro B , Kudo M , Kelley RK , Tak WY , Gasbarrini A , Reig M , Lim HY , Tougeron D , De Toni EN , Tam VC , Mody K , et al. Outcomes by occurrence of immune-mediated adverse events (imAEs) with tremelimumab (T) plus durvalumab (D) in the phase 3 HIMALAYA study in unresectable hepatocellular carcinoma (uHCC). J Clin Oncol. 2023; 41:4004. 10.1200/JCO.2023.41.16_SUPPL.4004. 37207300

[10] Celsa C , Cabibbo G , Fulgenzi CAM , Scheiner B , D’Alessio A , Manfredi GF , Nishida N , Ang C , Marron TU , Saeed A , Wietharn B , Pinter M , Cheon J , et al. Characteristics and outcomes of immunotherapy-related liver injury in patients with hepatocellular carcinoma versus other advanced solid tumours. J Hepatol. 2024; 80:431–42. 10.1016/j.jhep.2023.10.040. 37972660

[11] Xu S , Lai R , Zhao Q , Zhao P , Zhao R , Guo Z . Correlation Between Immune-Related Adverse Events and Prognosis in Hepatocellular Carcinoma Patients Treated With Immune Checkpoint Inhibitors. Front Immunol. 2021; 12:794099. 10.3389/fimmu.2021.794099. 34950153 PMC8691363

[12] Ng KYY , Tan SH , Tan JJE , Tay DSH , Lee AWX , Ang AJS , Wong LWJ , Choo SP , Tai DW , Lee JJX . Impact of Immune-Related Adverse Events on Efficacy of Immune Checkpoint Inhibitors in Patients with Advanced Hepatocellular Carcinoma. Liver Cancer. 2021; 11:9–21. 10.1159/000518619. 35222504 PMC8820151

[13] Galle PR , Foerster F , Kudo M , Chan SL , Llovet JM , Qin S , Schelman WR , Chintharlapalli S , Abada PB , Sherman M , Zhu AX . Biology and significance of alpha-fetoprotein in hepatocellular carcinoma. Liver Int. 2019; 39:2214–29. 10.1111/liv.14223. 31436873

[14] Piñero F , Anders M , Bermudez C , Demirdjian E , Varón A , Palazzo A , Rodriguez J , Beltrán O , da Fonseca LG , Ridruejo E , Caballini P , Tamagnone N , Reggiardo V , et al. Liver decompensation is a frequent cause of treatment discontinuation and prognostic factor in intermediate-advanced HCC. Ann Hepatol. 2023; 28:101110. 10.1016/j.aohep.2023.101110. 37100385

[15] Singal AG , Llovet JM , Yarchoan M , Mehta N , Heimbach JK , Dawson LA , Jou JH , Kulik LM , Agopian VG , Marrero JA , Mendiratta-Lala M , Brown DB , Rilling WS , et al. AASLD Practice Guidance on prevention, diagnosis, and treatment of hepatocellular carcinoma. Hepatology. 2023; 78:1922–65. 10.1097/HEP.0000000000000466. 37199193 PMC10663390

[16] European Association for the Study of the Liver. EASL Clinical Practice Guidelines: Management of hepatocellular carcinoma. J Hepatol. 2018; 69:182–36. 10.1016/j.jhep.2018.03.019. 29628281

[17] von Elm E , Altman DG , Egger M , Pocock SJ , Gøtzsche PC , Vandenbroucke JP , and STROBE Initiative. The Strengthening the Reporting of Observational Studies in Epidemiology (STROBE) statement: guidelines for reporting observational studies. Lancet. 2007; 370:1453–57. 10.1016/S0140-6736(07)61602-X. 18064739

[18] Johnson PJ , Berhane S , Kagebayashi C , Satomura S , Teng M , Reeves HL , O’Beirne J , Fox R , Skowronska A , Palmer D , Yeo W , Mo F , Lai P , et al. Assessment of liver function in patients with hepatocellular carcinoma: a new evidence-based approach-the ALBI grade. J Clin Oncol. 2015; 33:550–58. 10.1200/JCO.2014.57.9151. 25512453 PMC4322258

[19] Pugh RN , Murray-Lyon IM , Dawson JL , Pietroni MC , Williams R . Transection of the oesophagus for bleeding oesophageal varices. Br J Surg. 1973; 60:646–49. 10.1002/bjs.1800600817. 4541913

[20] Cancer Institute N. Common Terminology Criteria for Adverse Events (CTCAE) Common Terminology Criteria for Adverse Events v4.0 (CTCAE). 2009. https://www.eortc.be/services/doc/ctc/CTCAE_4.03_2010-06-14_QuickReference_5x7.pdf.

