# Supplementary material for: Immune-mediated adverse events following atezolizumab and bevacizumab in a multinational Latin American cohort of unresectable hepatocellular carcinoma
Source: Oncotarget. 2025 May 19;16:348–60. doi: 10.18632/oncotarget.28721 (PMC12088043; doi:10.18632/oncotarget.28721)
Supplement: Supplementary file 1 [file oncotarget-16-28721-s001.pdf]

Immune-mediated adverse events following atezolizumab and bevacizumab in a multinational Latin American cohort of unresectable hepatocellular carcinoma

SUPPLEMENTARY MATERIALS

Supplementary Table 1: Adverse events and immune related adverse events

Type of AE	Number of patients
**Non irAEs** **Total**	58
Fatigue	22
Arterial hypertension	12
Bleeding	11
Diarrhea	3
Rash	3
Hand-foot skin reaction	2
Proteinuria	2
Diabetes	1
Others	35
**irAEs** **Total**	18
Hepatitis	6
Thyroiditis	5
Nephritis	3
Adrenalitis	2
Myositis	1
Neurological	1
Others	1

Abbreviations: irAE: immune-related adverse events.
